# Can ‘Best Interests’ derail the trolley? Examining withdrawal of clinically assisted nutrition and hydration in patients in the permanent vegetative state

**DOI:** 10.1136/medethics-2015-103045

**Published:** 2016-08-31

**Authors:** Zoe Fritz

**Keywords:** Attitudes Toward Death, Autonomy, Ethics, Legal Aspects, Right to Refuse Treatment

## Abstract

In this paper, I explore under what circumstances it might be morally acceptable to transplant organs from a patient lacking capacity. I argue, with a developed hypothetical based around a mother and son, that (1) ‘Best interests’ should be interpreted broadly to include the interests that people have previously expressed in the well-being of others. It could, therefore, be in the ‘best interests’ of an unconscious patient to donate a non-vital organ to a family member. (2) Further expanding upon this case, and developing a variation on the ‘trolley problems’ I argue that where it is inevitable that an incapacitous patient is going to die—and specifically when it has been agreed through the courts that a patient in a permanent vegetative state is going to have clinically assisted nutrition and hydration withdrawn (with the inevitable consequence of death, and causing desiccation of the organs such that they are no longer able to be donated)—it could be in a patient's best interests to actively end their life with a drug that would stop the heart both to minimise potential suffering and in order to be able to have vital organs donated. I argue that in this case the strict adherence to the distinction between acts and omissions is not in the patient's best interests and should be reconsidered.

## Mother and son: part 1

Music blaring through earphones, a teenager stoops to pick something off the road and fails to notice the oncoming vehicle, or his mother's warning calls. His mother cannot stand by and watch him being crushed: she rushes onto the road to try to rescue him. In doing so, she puts herself in harm's way.

They are both injured: he goes into shock and renal failure. He has a congenital horseshoe kidney and requires a transplant. The mother—also injured, but not as badly—offers her own kidney, matches and donates it to her son.

No one would question her right to jump in front of the truck to protect her son, or to offer to donate her kidney; she can autonomously choose to put the life of her son before her own and to donate part of her body to him. It is accepted that, in determining what is in our best interests, we often consider the welfare of those around us: this is most starkly self-evident when considering the welfare of our children.

Now take a less happy scenario than the one above.

It starts as before, with the mother jumping in front of the vehicle. But this time, the mother's physical injuries were less than her son's but her brain injuries were more profound. She has lost capacity, although there is still hope that she will ultimately recover her brain function. No other family member matches her son for a transplant. Let us imagine, for the sake of this hypothetical, that dialysis is not an option. Her husband states that he knows his wife would want to donate a kidney to their child and asks that this be done.

## Best interests and substituted judgment

‘Best interests’ were enshrined in statute in the Mental Capacity Act in 2005,^1^ having previously been established in common law.^2–5^ To determine the ‘best interests’ of a person lacking capacity, the law requires that a process is undertaken which includes consider[ing], so far as is reasonably ascertainable:

- (1) The person’s past and present wishes and feelings (and, in particular, any relevant written statement made by him when he had capacity)
- (2) The beliefs and values that would be likely to influence his decision if he had capacity
- (3) The other factors that he would be likely to consider if he were able to do so.^1^

In a recent judgment (Aintree vs James)^6^ Lady Hale emphasised that, in determining ‘best interests’ one must

 look at welfare in the widest sense, not just medical but social and psychological; ….try and put themselves in the place of the individual patient and ask what his attitude to the treatment is or would be likely to be; and…consult others who are looking after him or interested in his welfare, in particular for their view of what his attitude would be.

A false dichotomy has arisen distinguishing ‘best interests’ OR ‘substituted judgment’. Substituted judgment is used widely in the USA,^7^ is grounded in respect for autonomy and instructs the surrogate to make a decision that the person would have made if they had capacity.

Problems with the ‘substituted judgment’ test have been highlighted^8^ and, in particular, it has been emphasised that surrogates are not always accurate predictors of what someone would have wanted.^9^

Ideally—as in Hale's judgment—determining best interests *includes* an assessment of ‘substituted judgment’, as part of an objective process to determine what is in the patients best interests, while not granting overriding authority to the opinions of those close to the patient.

In the case of *Re X, Y and Z*,^10^ concerning a decision about whether some settlement money should be used for the care of the incapacitated patient's children: Baker J stated: “Where a parent loses mental capacity at a time when she is still responsible for her children, those responsibilities are part of her ‘interests’ which have to be addressed by those making decisions on her behalf.” He ruled that the money could be spent on her children's nanny, thus reducing what was available for her own care. In doing so, he was acknowledging that the patient's best interests might include putting the interests of her children first.

In our hypothetical case, the mother has frequently said that she would always put her children first. She has demonstrated through her behaviour (jumping in front of the car) that she puts her son's health before her own.

## Can organ donation be in an incapacitated individual's ‘best interests’

If a ‘best interests’ decision really includes ‘best interests’ in a wider sense—including the welfare of her family and specifically that of her son—would removing her kidney to benefit her son be the correct thing to do? She would continue living (with only one kidney), with a similar chance of recovery from her brain injury, and will save her son. To not carry out the transplant is in neither of their best interests: imagine the mother surviving her injury, regaining consciousness and discovering that her son had died because the family was not sure that she would have wanted her kidney to be donated.

There is, however, no legal precedent in the UK for organ donation from an incapacitated patient. There has been at least one case of a bone marrow transplant being approved in Re Y,^11^ but the judge noted that this was an unusual case and that it depended on three facts: the procedure was very low risk, there was no evidence that Y objected and there was some plausible benefit to Y. Connell J explicitly stated that the case would not be a useful precedent in cases involving serious surgery such as organ donation.

Is there a context in the UK where whole organ donation from an incapacitated patient might be allowed? The precedent was allowed in the USA in 1972,^12^ where a 7-year-old identical twin was allowed to donate her kidney to her sister.

In the mother and son case of donating a kidney, there would be some physical risk to the mother in making the donation, but she would not be sacrificing her life, and there would be more than plausible benefit from her donation. Following these arguments, the courts might therefore approve the mother donating her kidney, even when incapacitated.

There are, however, limits to how much the mother can go on protecting her son once she loses capacity. A safeguard was built into the Mental Capacity Act which implicitly puts the sanctity of life above the principle of acting in the patient's best interests: ‘If the decision concerns life-sustaining treatment, a person trying to work out the best interests of a person who lacks capacity should not be motivated in any way by a desire to bring about the person's death’.^1^

## Mother and son: part 2

So let us take the scenario in a different direction. The son has physical injuries from which he makes a good initial recovery, but needs continued medication for pain relief and ongoing infections. The mother sustained a catastrophic brain injury which leaves her in a vegetative state (VS). Time passes and the mother does not make any neurological recovery, although her body is otherwise well. At a year, her family is told that she is in a permanent VS (PVS); clinicians are unanimous that she is unlikely to ever recover any consciousness. She receives clinically assisted nutrition and hydration (CANH) through a tube which was placed shortly after her injury. She requires 24-hour care in a nursing home, with specialised support to prevent contractures and other complications of her condition. A ‘best interests’ meeting is held, and the family is unanimous in their belief that she would not want to continue in this state; an application is made to the court of protection for withdrawal of CANH.

A year later, her case is heard. The judge listens to her husband, son and daughter, and evidence from her nursing colleagues on the transplant ward on which she had worked, part time, for many years. All agree that she would not want to live like this. Beyond the arguments that have been made in other such cases—that she would have found it undignified and that she wouldn't have wanted to exist as a body alone—there is evidence provided that to continue like this could not be in her best interests in the widest sense. She was, the judge hears, an extraordinarily selfless and generous person, and one of the things which would have troubled her was the idea that her family's life was now centred around her, despite her lack of consciousness. Her children and husband continue to visit her every weekend; Christmas has been spent by her bedside. Her friends and colleagues further say that she would have been uncomfortable with the idea of the state having to pay in the region of 100 000 yearly^13^ when that money could have been spent on other patients who had a chance of recovery. Both family and friends present a case that it would not be in her best interests to continue like this because her best interests lie in her own medical welfare and in the welfare of those around her.

The judge listens to expert witnesses about her diagnosis and prognosis. She is now 2 years post her initial injury, with no sign of recovery. She has had multisensory assessments over several periods which suggest that she is completely insentient. She has participated in research into functional MRI and EEG, which show no suggestions of covert consciousness.

The precedent for withdrawing treatment including CANH in a patient in a VS was made in the UK in 1992 in the case of Tony Bland.^14^ In the judgment it was stated: “the question is not whether it is in the best interests of the patient that he should die. The question is whether it is in the best interests of the patient that his life should be prolonged by the continuance of this form of treatment … I cannot see that medical treatment is appropriate or requisite simply to prolong a patient's life when such treatment has no therapeutic purpose of any kind, as where it is futile because the patient is unconscious and there is no prospect of any improvement in his condition.” Consistent with this, it has been agreed that on the death certificates of those patients in whom CANH is withdrawn, the cause of death is recorded as the original acute brain injury, not starvation.

Returning to our case: while the judge is reviewing the evidence as to whether it is in the mother's best interests to withdraw CANH, the son continues on multiple courses of antibiotics and pain relief for associated complications from his injury. After the case is heard (but before the judgment has been issued), the son goes into acute liver failure: it is thought that this is secondary to the medications he has been taking since the accident. He is admitted to intensive care and listed superurgently for a liver donor. The day later, the judgment is published: it is not in the mother's best interests to continue with CANH and it should be withdrawn. She will die (with certainty) within about 3 weeks.

The father asks the doctors: “if my wife matches our son, why don't we give him her liver?”

In order for the liver to be able to be used for the son, the mother would need to die quickly—waiting for her to die from starvation and dehydration would result in desiccated and severely damaged organs. The only way for her to die quickly would be for her to actively end her life with a drug that would stop her heart, and then donate her organs; the liver for her son, and the others for those who need them; to actively sacrifice her—almost ended—life for others. The most famous thought experiments around this kind of decision are the ‘Trolley problems'.

## The ‘trolley problems’

The ‘trolley problems’ were first conceived of by Phillippa Foot^15^ and expanded upon by Judith Jarvis Thomson.^16^ In the first, a trolley is heading down a track, which divides. It is heading to the left, where five workers are on the rail: if it continues, all five will be killed. If the driver moves the course of the trolley to the right, it will kill only one worker. A variant on this is to have a bystander who can flick a switch and change the tracks.

Most people, when surveyed, would be comfortable flicking the switch or changing the trolley such that they are responsible for one death rather than allowing five to die.^17^ People become less comfortable, however, if the death of that one is not as a consequence of saving the five (flicking the switch or moving the trolley to avoid the five) but rather as a means by which the other five are saved. One example of this is where there is a fat man peering over the railings of a bridge—he could be pushed over, onto the tracks, to stop the trolley and thus save the five. (In this scenario, you yourself are too slim to even slow the trolley—you would just die needlessly. The only possibility of saving the five is to push the fat man.) Another example—which removes the personal proximity of pushing the man—is if the trolley was on a loop, on which lay one individual whose death would slow the trolley sufficiently that the other five could escape.

In all these cases, the discussion centred around acts versus omissions^18^ (allowing the trolley to go on its direct intended route killing the five rather than taking responsibility for the death of the one); around utilitarian arguments (it is better to save five rather than one) and, with the loop/fatman scenarios on using a person as a means to prevent a death rather than as an end result of saving someone else.

To build on these, I would like to put forward two more ‘trolley problems’ which relate to the story I have described above. The first is that of the paraplegic (but fully competent) mother on the bridge.

She sees her son with others on the tracks, sees the trolley coming and begs you to throw her over the rails to save the people. Her disability precludes her from doing it herself; she needs your assistance. She would be the means of saving the five, and she is competent to request it; you, however, would be assisting her suicide, as a means—requested by her—to save others. To refuse her request, to stand by and watch her watch her son being killed, would be to deprive her of her autonomous, capacitous choice. By acceding to her wish, you are enabling her to have the same rights as the mother who threw herself in front of the truck in the first scenario.

**Figure 1:**
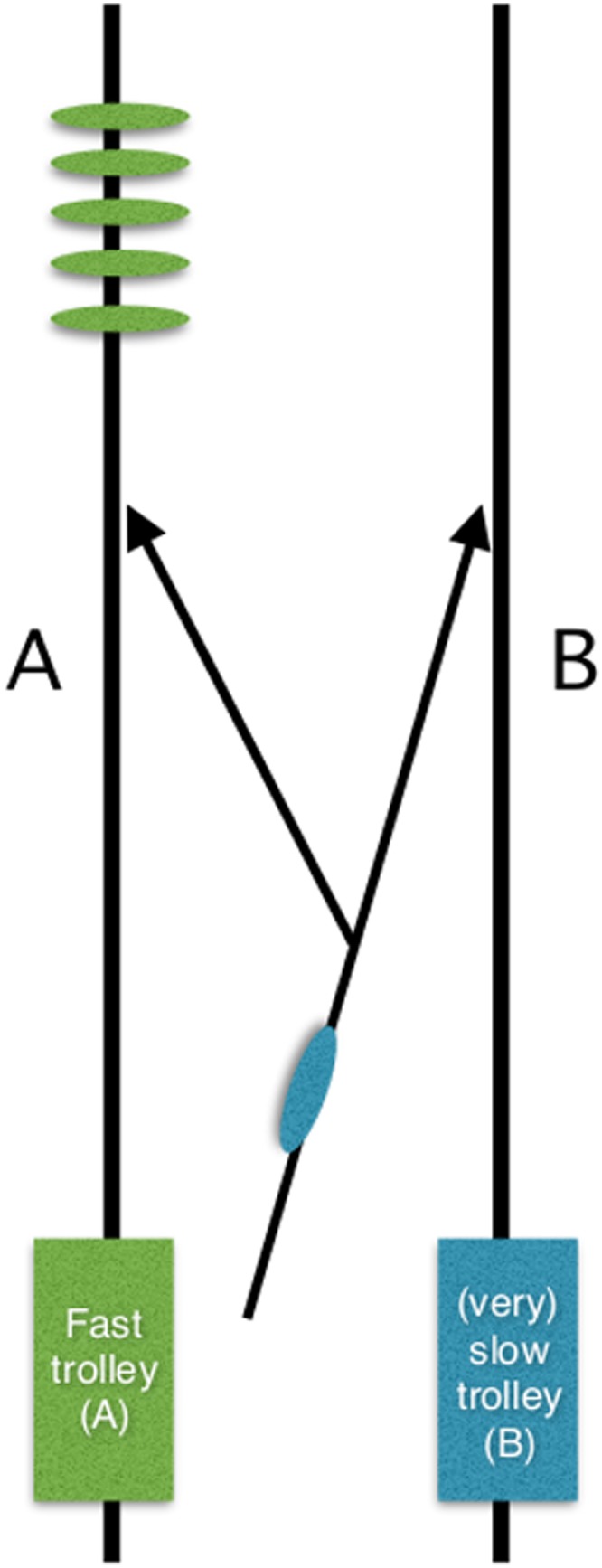
‘Trolley problem’ illustration: lady in whom Court of Protection has agreed clinically assisted nutrition and hydration (CANH) should be withdrawn on bifurcating ramp currently heading towards empty track with a slow train approaching; 5 others are on parallel track with fast train approaching.

A final ‘trolley’ problem involves a patient without capacity. Specifically, a patient in a PVS, for whom a decision has already been reached, through the courts, that CANH should be withdrawn. A patient who has shown by her own actions as well as previously stated wishes that she would put her son's life before that of her own. This time there is a Y-shaped ramp leading down to parallel trolley tracks, and there are two trolleys (A and B) going along the tracks: trolley B is heading slowly towards an empty track and trolley A is heading quickly towards the son and four others (see figure 1). The patient in the PVS is rolling towards the empty track—to do nothing will mean that she is injured by trolley B, dying in 3 weeks, while, on the parallel track, her son and four others are going to be killed by trolley A. To move her on the ramp would mean that she is killed instantly by trolley A, but it would be derailed, and her son and four others would survive; our action would make her the means to their being saved. Would this be in her wider best interests?

## Death via withdrawal of treatment versus death via an act

The sharp delineation between withdrawing treatment with the inevitable consequence of death and causing death by an act is enshrined in law: it is legal—with the appropriate judicial review as described above—to cease to feed a patient when we believe that it is not in their ‘best interests’, with the inevitable consequence of death. These ‘best interests’ decisions tend to be based around the patient's lack of sentience, and the belief that they would not want to live in an undignified manner or incapacitated state. It is not often argued on medical grounds that giving CANH is detrimental to them: a well-established percutaneous endoscopic gastrostomy (PEG) feed has very few complications.^19^ The withdrawal of CANH, on the other hand, causes physiological responses which are, at the very least, unpleasant for those caring for the patient to witness.^20^

It is recommended that when CANH is withdrawn it is done so with specialist palliative care input, and with all medications which are normally prescribed to alleviate suffering—opiates, sedatives, etc.^20^ ‘Terminal sedation’—the prescription of such drugs to alleviate unbearable suffering, with the potential of hastening death via the doctrine of double effect—has long prompted ethical debate.^21^ The line between ‘terminal sedation’ and euthanasia is a thin one, which becomes even less well defined when the patient is considered unable to experience the very symptoms which the drugs are designed to alleviate. French lawmakers, following the recent decision in Lambert versus France,^22^ are considering legislation requiring that CANH be withdrawn only under ‘deep sedation’.

So back to our hypothetical case, the father is asking whether his dying wife's healthy liver might be transplanted into his otherwise dying son. The mother is going to die: it has been agreed by the courts, in a carefully regulated manner, that she is not benefiting from her CANH and that it should be stopped. Her leaving of life in this manner will be slow and will preclude her organs being donated.

The court approval (at least 100 times in the UK since Bland) of patients in PVS having CANH withdrawn, rather than their lives actively ended shows us as a society which draws a clear distinction between acts and omissions. Although the possibility of organ donation has not been considered in these judgments we can extrapolate that this would not be countenanced either: as a society, we would be afraid of actively moving the (insentient, dying) patient away from the slow inevitable death on track B to save the five on track A who could benefit from the organs—even if that was in accordance with the patients previously stated wishes. The law is clear that there are no circumstances in which euthanasia is legal.

In the Bland ruling, Lord Browne-Wilkinson said:

 …the conclusion I have reached will appear to some to be almost irrational. How can it be lawful to allow a patient to die slowly… but unlawful to produce his immediate death by a lethal injection..? I find it difficult to find a moral answer to that question. But it is undoubtedly the law.^14^

The status quo—of allowing withdrawal of CANH with the inevitable consequence of death, while forbidding actively ending life with a drug that would stop the heart—is an ethical fudge, following the law, rather than making it.

## ‘Organ donation euthanasia’

In the case I have developed, I argue that allowing the mother to donate her liver to her son would be acting in her best interests; at a point where she herself has nothing to gain from her organ, why would we prevent the donation of it to the son she was trying to save? The case for ‘organ donation euthanasia’ (ODE) has been made by Wilkinson and Savulescu.^23^ They argued that doing so in a patient in whom it has been decided to withdraw life-sustaining treatment would be a Pareto^24^ improvement: ‘In all cases the patient dies, but in the case of ODE more lives are able to be saved by harvesting functioning organs, and the desire of the patient that their organs be used to help others is more likely to be able to be respected’. The argument for ODE was recently added to by Lazaridis and Blumenthal-Barby.^25^ In particular, they stipulate safe guards to ensure that the donor would not be worse off or harmed in other ways and argue that ODE would not be treating such patients as ‘means’: ‘We are not regarding patients who undergo withdrawal of life support and organ donation merely as a means when we minimize, or in fact eliminate, the suffering associated with the dying process and when we allow for their end-of-life preferences to be accommodated’. Using this argument, the PVS mother on the ‘ramp’ benefits dually in being ‘diverted to track A’; she is not only acting as a means to save her son, but would be having the potential of her own suffering eliminated.

Many would not believe it was right to stand by on the bridge beside the woman and watch the trolley kill her son. But even when the case is less stark—in cases of withdrawal of CANH in patients in VS more generally—there are moral inconsistencies. We currently defend a total ban on euthanasia even when using it might minimise suffering for the individual (swift death as opposed to potential suffering from withdrawal of CANH) and for those in need of organs.

The strongest argument against legalising euthanasia in this circumstance is that, by making it an absolute—it is never right to wilfully end a life—we are protecting society both from a ‘slippery slope’ of euthanasia in less regulated situations and an erosion of its moral framework. But this is a position that is upheld by fear, and which tolerates the suffering of a real (very) few because of concerns about some other (hypothetical) few and a possible societal shift towards tolerating euthanasia more generally.

In the final trolley problem above, the utilitarian argument would be that the patient in PVS should be diverted towards the track to save the five in all circumstances, in accordance with previous wishes. However, to skip the step of working out what the individual would have wanted, to ignore the possibility of considering their autonomy even when they lack capacity would be to reduce them to a body with organs rather than respecting them as a human.

Conversely, in the circumstance of a patient in PVS who has been through the court of protection, where it has been agreed that CANH is not in their best interests, it should be morally and legally permissible—or even mandated—to explore what is in their best interests in the wider sense, what their wishes were in terms of organ donation and what means of dying they would prefer. If the conclusion from this consideration—in the courts, with appropriate safeguarding—is that they would wish their life to be actively ended facilitating the donation of their organs, then this should be respected.

## Conclusion

(1) ‘Best interests’ should include the interests that people have previously expressed in the well-being of others; this extends to altruistic deeds. It could therefore be in the best interests of an unconscious patient to donate a non-vital organ to a family member. (2) Where it is inevitable that an incapacitous patient is going to die—and specifically when it has been agreed through the courts that a patient in a PVS is going to have CANH withdrawn, it could be in a patient's best interests to have a drug that would stop their heart and to have vital organs donated to a family member, acting as a means to the end of saving another, much as the mother would be doing in running out on the road to save her son. By extension, it could also be in the patients best interests to donate their organs to someone else, if that was consistent with their previously expressed wishes. (3) The current practice of withdrawing CANH from patients in PVS or minimally conscious state—with the inevitable consequence of death—is ethically inferior to actively ending life with a drug that would stop the heart. The ‘act’ rather than the omission would allow families to be present at the death of their loved one and obviate the potential for the physiological signs seen with starvation and dehydration; it would negate the (although tiny) possibility that the individual suffers during withdrawal of CANH, rather than addressing this with sedation and analgesia as is currently the case. And it would allow those who had previously expressed a desire to donate their organs to do so allowing their altruistic desires to be respected as part of a wider interpretation of best interests.
